# Supplementary figures and images for: Zero-shot style transfer for gesture animation driven by text and speech using adversarial disentanglement of multimodal style encoding
Source: Front Artif Intell. 2023 Jun 12;6:1142997. doi: 10.3389/frai.2023.1142997 (PMC10291316; doi:10.3389/frai.2023.1142997)

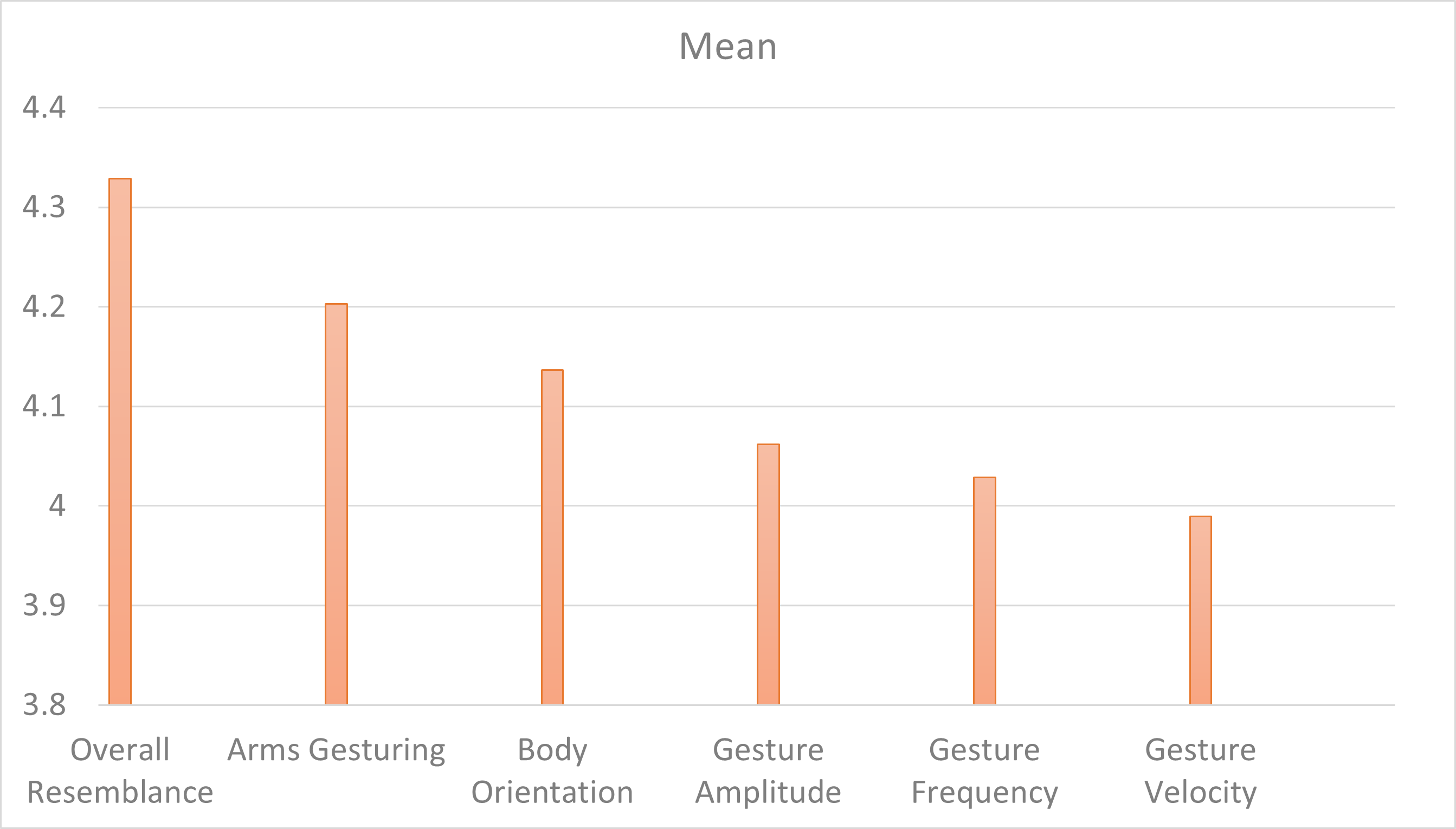

Supplement: Supplementary file 2 [file Data_Sheet_2.ZIP › Submission_FIAI_DataSheet2/ZSMSTMSeen.png]

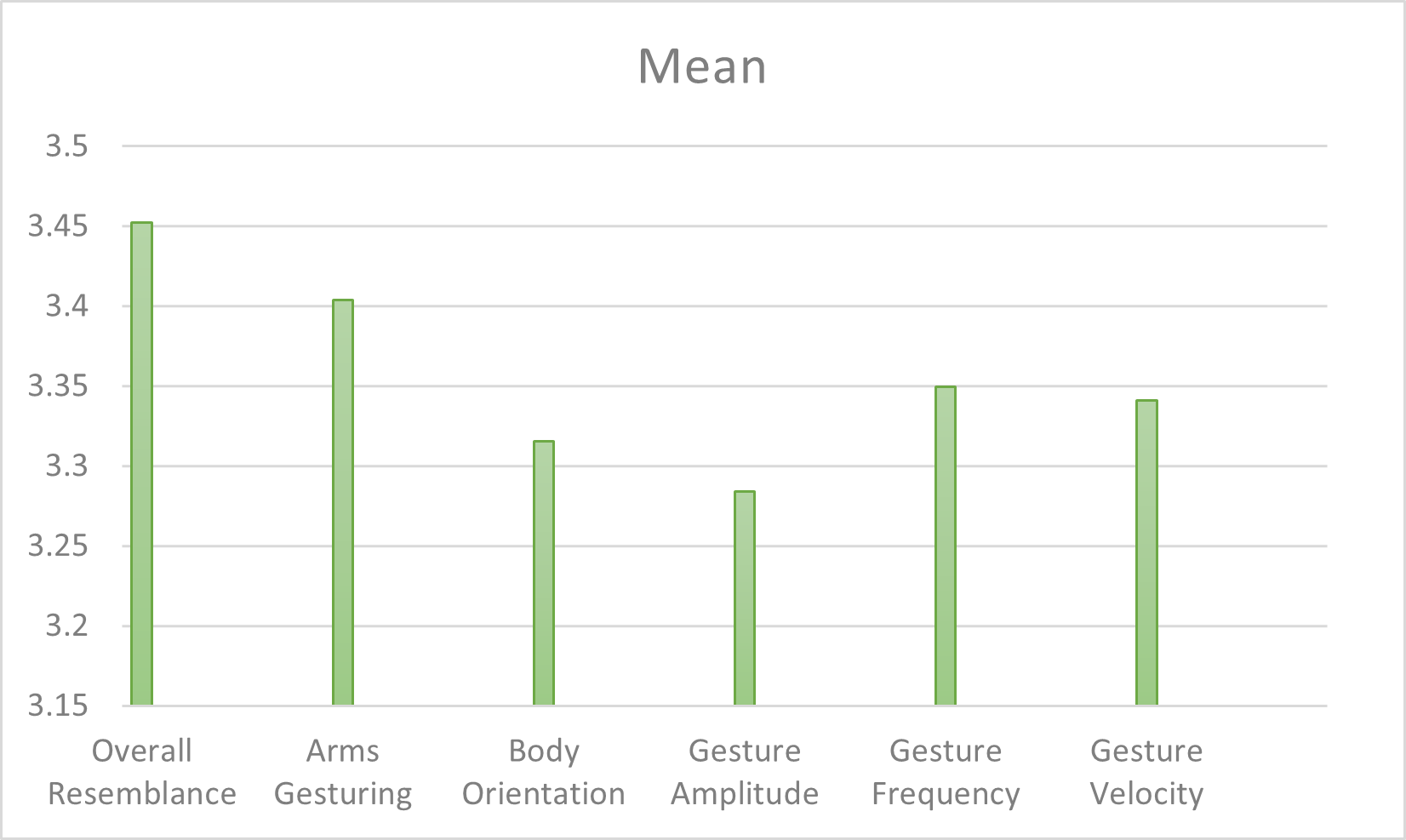

Supplement: Supplementary file 2 [file Data_Sheet_2.ZIP › Submission_FIAI_DataSheet2/ZSMSTMUnseen.png]
